# Chemistry of the M (M=Fe, Ca, Ba)-Se-H_2_O Systems at 25 °C

**DOI:** 10.3390/molecules14093567

**Published:** 2009-09-14

**Authors:** Tadahisa Nishimura, Ryosuke Hata, Fumihiko Hasegawa

**Affiliations:** 1 Institute of Multidisciplinary Research for Advanced Materials (IMRAM), Tohoku University, 1,1 Katahira, 2-chome, Aobaku, Sendai 980-8577, Japan; 2 Graduate School of Student, Tohoku University, Currently Ebra Co. Ltd., Tokyo, Japan; E-Mail: ryoplace@pop01.odn.ne.jp (R.H.); 3 New Industry Creation Hatchery Center Tohoku University, Aoba, Aramaki, Aobaku, Sendai 980-8579, Japan; E-Mail: hasegawa@niche.tohoku.ac.jp (F.H.)

**Keywords:** aqueous chemistry, ferric selenite, calcium selenite and selenate, barium selenite and selenate, removal, selenium

## Abstract

The chemistry of the M (M=Fe, Ca, Ba)-Se-H_2_O systems at 25 °C is reviewed based on our previous papers. In this paper, the phase equilibria in the Fe(III)-Se(IV)-H_2_O, Ca-Se(IV,VI)-H_2_O and Ba-Se(IV,VI)-H_2_O systems at 25 °C are discussed. Then, the three-stage process for removal of selenium from industrial waste water [Se(IV,VI) < 1,500 mg/L] containing sulfuric acid was introduced. This seems to be a promising process for selenium removal from acidic sulfate waste water containing high concentration levels of selenium to below 0.1 mg/L.

## 1. Introduction

Selenium, atomic number 34, is a member of the Group16 elements together with oxygen, sulfur, tellurium and polonium. The most relevant oxidation states of selenium in aqueous solution are −2, 0, +4 and +6. Selenium(-II) species (H_2_Se, HSe^−^ and Se^2−^) are fairly rapidly oxidized to elemental selenium in air. Both selenium(IV) species (H_2_SeO_3_, HSeO_3_^−^and SeO_3_^2−^) and selenium(VI) species (H_2_SeO_4_, HSeO_4_^−^ and SeO_4_^2−^) are predominant in aqueous solutions, depending on the oxidation-reduction potential and pH of the solution. 

Selenium is also a significant component in ores such as chalcopyrite, galena and pyrite. In smelting of copper and lead, the majority of selenium is concentrated in the anode slime formed during electrolytic refining of copper, but significant quantities are followed in the sludge accumulating in sulfuric acid plants and in the electrostatic precipitator dust collected during the processing of ores and concentrates. These three intermediate products have been processed for recovering some valuable metals and selenium by the pyrometallurgical and/or hydrometallugical processes. The selenium-containing waste waters resulting from these processes should be treated in an environmentally acceptable manner. Five detailed reviews of the work on the removal of selenium(IV) and selenium(VI) from waste water were published by Koren *et al.* [1], Kapoor *et al*. [2], Mirza and Ramachandran [3] and Hata *et al.* [4] and Twidwell *et al.* [5], in which most works cited deal with precipitation, coprecipitation, adsorption, ion exchange, reverse osmosis elimination, chemical reduction and biological reduction of selenium entities. These processes are applicable to the removal of selenium(IV) from waste water, but not very effective to remove selenium(VI) under mild conditions. Hydrazine can reduce selenium(VI) to elemental selenium only in hot and concentrated sulfuric acid solution [6]. Although some anaerobic bacteria are capable of reducing selenium(VI) to elemental selenium[7,8,9,10,11], a weakness of this technique is that it requires days rather than minutes for the reduction.

An aqueous chemistry of selenium is closely related to the treatment of selenium-bearing waste waters and the hydrometallurgical processing of selenium-bearing intermediates. In this paper the chemistry of the M (M=Fe, Ca, Ba)-Se-H_2_O systems at 25 °C is reviewed based on our papers [12,13,14], and then a three-stage process for removal of selenium from industrial waste water [Se(IV,VI) < 1,500 mg/L) containing sulfuric acid to below 0.1 mg/L is discussed [15,16]. The BaSO_4_ method used in the second stage allows removal of selenium(VI) to a level of around 2 mg/L. In order to remove selenium(VI) below 0.1 mg/L, a modification of the reduction method of selenium(VI) to elemental selenium by ferrous hydroxide in alkaline solution developed by Murphy [17] was successful, and furthermore, the use of the polyamine-type weakly basic ion exchange resin (Eporasu K-6) was attempted.

## 2. Chemistry of the M (M=Fe, Ca, Ba)-Se-H_2_O Systems at 25 °C

### 2.1. Equilibrium in the Fe(III)-Se(IV)-H_2_O System [12]

**Figure 1:**
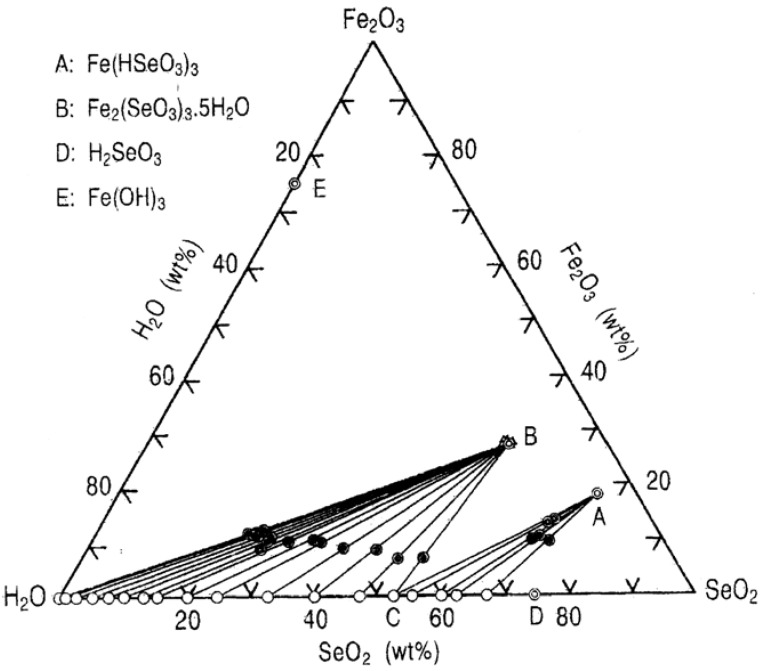
Ternary phase diagram for the Fe_2_O_3_-SeO_2_-H_2_O System at 25 °C.

The Fe_2_O_3_-SeO_2_-H_2_O system is interesting in relation to the removal of selenium(IV) from hydrometallurgical solutions by the precipitation with certain ferric species. Selenious acid solutions suspended with the starting precipitate, i.e., amorphous ferric selenite, were mechanically shaken in an air bath kept at 25 °C for three months to allow equilibrium to be established between the solid and liquid phases. The results from the analysis of solutions, wet residues and precipitates (dried in vacuum) for 30 different runs were converted to weight percent values to enable the construction of the ternary phase diagram for the Fe_2_O_3_-SeO_2_-H_2_O system, which is shown in Figure 1. In this figure, the compositions of solution, wet residue and precipitate are given by ○, ● and Δ, respectively. The graphical procedure based on the wet residue method proves the existence of two solid phases of Fe(HSeO_3_)_3_ and Fe_2_(SeO_3_)_3_∙5H_2_O indicated by the points A and B. At the invariant point C (Fe_2_O_3_ = 0.02 wt%, SeO_2_ = 52.4 wt%), the two solid phases are in equilibrium. Point D (SeO_2_ = 74.7 wt%) represents a saturated solution of selenious acid and is the equilibrium point between Fe(HSeO_3_)_3_ and solid selenious acid. The Fe_2_O_3_ concentration of the liquid phase is very low, and it is approximately 0.02 wt%, even at the invariant point C.

The X-ray diffraction (XRD) patterns for compound A, Fe(HSeO_3_)_3_, did not match with any compound in the International Center for Diffraction Data (ICDD) Cards, whereas the XRD patterns for compound B was similar to that in the data for the mineral mandarinoite, Fe_2_(SeO_3_)_3_∙4H_2_O, which is quoted in the ICCD Card No. 29–718. However, the chemical analysis of the solid phase of compound B indicated that the ferric selenite had a hydration number of 5, i.e., it is a pentahydrate rather than being mandarinoite. Also, the differential thermal analysis and thermogravimetric analysis conformed that a hydration number was 5.

**Figure 2:**
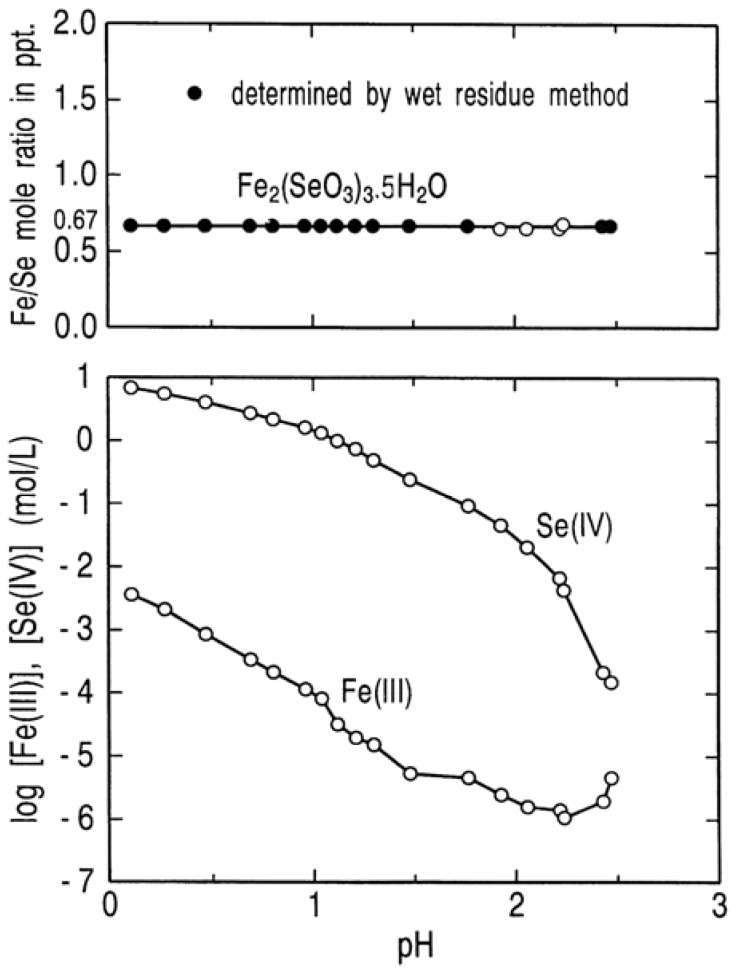
Stability and solubility regions for ferric selenite as a function of pH at 25 °C.

Figure 2 is a dual plot of Fe(III)/Se(IV) mole ratio in the solid phase and the log concentration data for the solution phase, both versus pH. These plots express the stability and solubility regions for ferric selenite as function of pH at 25 °C. It is clear from Figure 2(a) that Fe_2_(SeO_3_)_3_.5H_2_O is stable in the pH range 0.1 to 2.5 which was considered in our work. Figure 2(b) shows that the iron concentration in solution in equilibrium with Fe_2_(SeO_3_)_3_∙5H_2_O has a minimum value of 6.0 × 10^−5^ mol/L at pH 2.2, while the selenium concentration decreases monotonously with an increase in pH. Hence, it may be concluded that selenium(IV) in solution containing ferric ion is basically removed in the form of Fe_2_(SeO_3_)_3_∙5H_2_O compound under moderate conditions. 

### 2.2. Equilibrium in the Fe(III)-Se(VI)-H_2_O System 

The formation of ferric selenate at moderate temperature has been not reported. Only the XRD data for Fe_2_(SeO_4_)_3_∙9H_2_O which was synthesized from an aqueous solution of Fe and selenic acid by slow evaporation over a period of several months are listed in the ICDD Card No.45 – 544.

### 2.3. Equilibrium in the Ca-Se(IV)-H_2_O and Ca-Se(VI)-H_2_O Systems [13]

In the hydrometallurgical and mining waste water processes, calcium hydroxide and calcium carbonate have often been used as a neutralizing agent or pH adjustor. Sulfate ion is usually removed as CaSO_4_∙2H_2_O (gypsum) in nonferrous hydrometallurgy. Since selenium has similar properties to sulfur, it is possible for selenium to co-precipitate with gypsum. Also, selenium with sulfur may be incorporated into the sludge produced in the waste gas-desulfurization stage. The Ca-Se(IV)-H_2_O and Ca-Se(VI)-H_2_O systems are closely related to the treatment of selenium-contaminated process liquors in these hydrometallurgical applications and to the mineralogy relating to the occurrence of any calcium selenites and calcium selenates.

**Figure 3:**
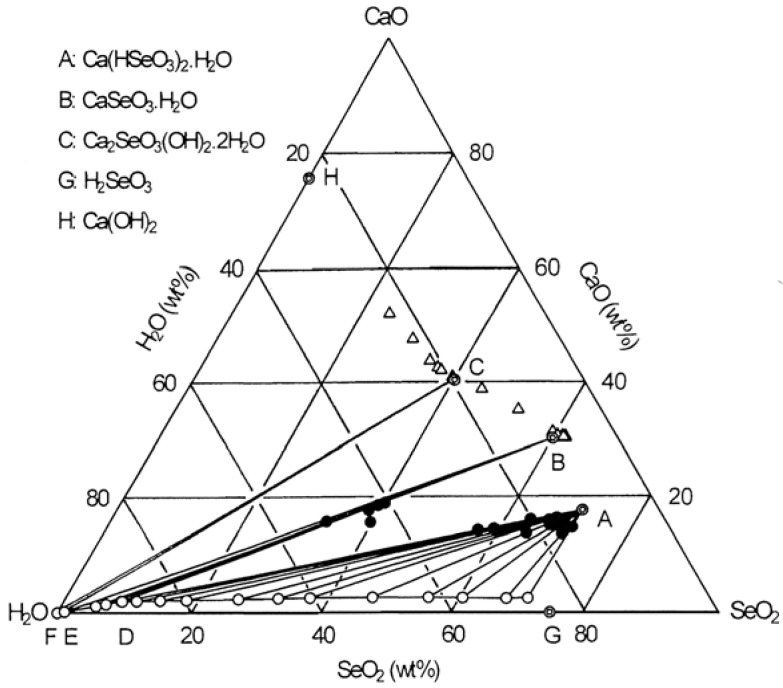
Ternary phase diagram for the CaO-SeO_2_-H_2_O system at 25 °C.

Selenious acid solutions containing a suspension of CaO were held under mild shaking for one month at 25 °C. The original analytical data for the solutions, wet residues and precipitates are presented graphically in Figure 3 as the ternary phase diagram for the CaO-SeO_2_-H_2_O system. It is obvious from this graphical procedure that two compounds, Ca(HSeO_3_)_2_∙H_2_O and CaSeO_3_∙H_2_O, denoted by A and B, exist. Also, the chemical analysis of three precipitates obtained at around pH = 12.0 gave a mole ratio of Ca/Se(IV) of 2.0, corresponding to the Ca_2_SeO_3_(OH)_2_∙2H_2_O species with CaO = 40.47 wt%, SeO_2_ = 40.04 wt% and H_2_O = 19.49 wt% (given by C). In this system, there are three invariant points. The first invariant point lies at point D (CaO = 2.1 wt%, SeO_2_ = 8.6 wt%) where Ca(HSeO_3_)_2_∙H_2_O and CaSeO_3_∙H_2_O coexist. The second point lies at point E (CaO = 0.06 wt%, SeO_2_ = 0.003 wt%) where CaSeO_3_∙H_2_O and Ca_2_SeO_3_(OH)_2_∙2H_2_O coexist. The third point lies at point F (CaO = 0.08 wt%, SeO_2_∙= 0.00004 wt%) where Ca_2_SeO_3_(OH)_2_·2H_2_O and Ca(OH)_2_ coexist. Point G (SeO_2_ = 74.7 wt%) represents a saturated solution of selenious acid. The stable regions are bounded for Ca(HSeO_3_)_2_∙H_2_O by ADG, for CaSeO_3_∙H_2_O by BED and for Ca_2_SeO_3_(OH)_2_∙2H_2_O by CFE. 

The XRD patterns for compounds A and B formed in high and low selenious acid solutions were identical to those in ICCD Cards No. 36 – 467 for Ca(HSeO_3_)_2_∙H_2_O and No. 35 – 883 for CaSeO_3_∙H_2_O, respectively. However, the XRD patterns for compound C, Ca_2_SeO_3_(OH)_2_∙2H_2_O, formed in alkaline solutions could not matched with any patterns in the ICCD Cards.

**Figure 4:**
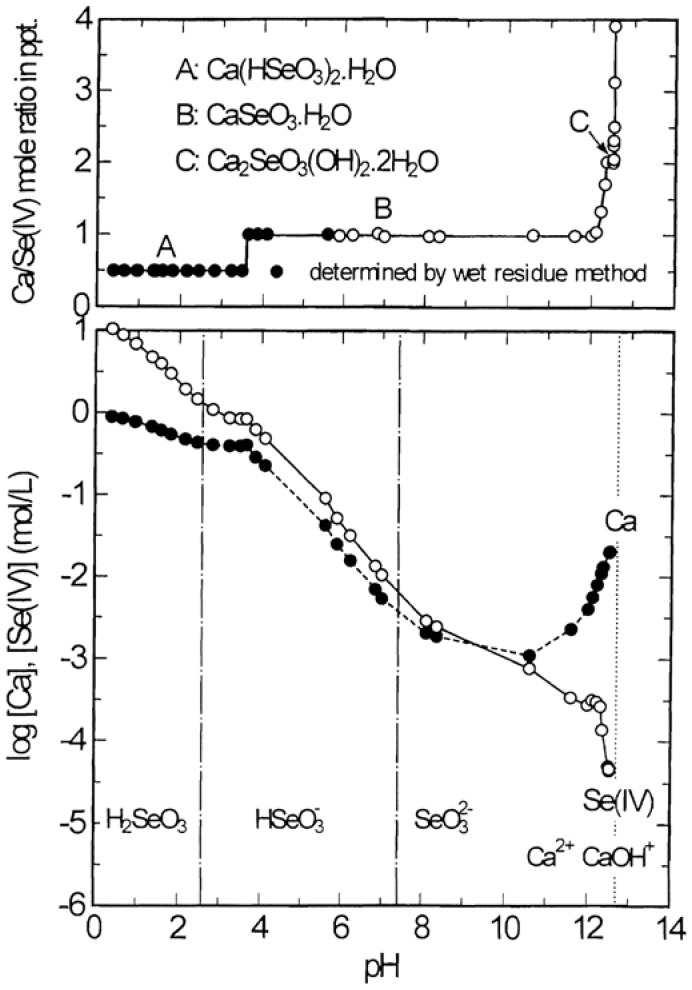
Stability and solubility regions for calcium selenites as a function of pH at 25 °C.

Figure 4 shows the stability and solubility regions for calcium selenites as function of pH at 25 °C. It is noted from Figure 4(a) that Ca(HSeO_3_)_2_∙H_2_O with Ca/Se(IV) mole ratio of 0.5 is stable at pH below 3.5, CaSeO_3_∙H_2_O with Ca/Se(IV) mole ratio of 1.0 in the pH range 3.5 to 12.3 and Ca_2_SeO_3_(OH)_2_∙2H_2_O with Ca/Se(IV) mole ratio of 2.0 in the pH range 12.3 to 12.5. In this system, there are three invariant points at pH 3.5, 12.3 and 12.5, at each point, two solids phases, Ca(HSeO_3_)_2_∙H_2_O and CaSeO_3_∙H_2_O, CaSeO_3_∙H_2_O and Ca_2_SeO_3_(OH)_2_∙2H_2_O, and Ca_2_SeO_3_(OH)_2_∙2H_2_O and Ca(OH)_2_ coexist. Figure 4(b) shows that the calcium concentration in solutions in equilibrium with the calcium selenites has a minimum value of 1.1 × 10^−3^ mol/L at pH 10.6, while the selenium(IV) concentration has a minimum value of 4.4 × 10^−5^ mol/L at pH 12.5 where Ca_2_SeO_3_(OH)_2_∙2H_2_O is in equilibrium with the solution of high calcium concentration. It should be noted that this lowest concentration for selenium (IV) (3.5 mg/L) is still much higher than the limit of industrial waste water regulation for selenium (0.1 mg/L) as specified by most regulatory authorities.

**Figure 5:**
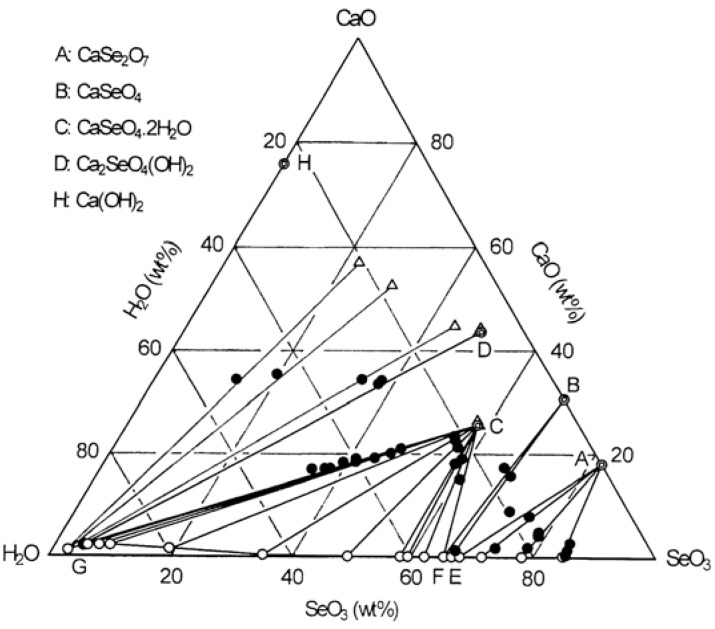
Ternary phase diagram for the CaO-SeO_3_-H_2_O system at 25 °C.

A ternary phase diagram for the CaO-SeO_3_-H_2_O system at 25 °C is shown in Figure 5. In this equilibrium experiment, selenate acid solution (which was prepared by dissolving H_2_SeO_3_ in distilled water) was used as the starting material. 

The diagram proves the existence of three compounds given by A, B and C. Also, the chemical analysis of three precipitates obtained at around pH = 12.0 showed a mole ratio of Ca/Se(V) of 2.0 corresponding to the Ca_2_SeO_4_(OH)_2_ with CaO = 43.62 wt%, SeO_3_ = 49.38 wt% and H_2_O = 7.00 wt% (given by D). From these results, it can be seen that there are four calcium selenates which are represented by the formulae CaSe_2_O_7_, CaSeO_4_, CaSeO_4_∙2H_2_O and Ca_2_SeO_3_(OH)_2_ and four invariant points, given by the points E (CaO = 0.09 wt%, SeO_3_ = 66.62 – 67.93 wt%), F (CaO = 0.09 wt%, SeO_3_ = 65.55 wt%), G (CaO = 2.20 wt%, 4.70 wt%) and H (CaO = 0.99 wt%, SeO_3_ = 1.95 wt%).

The XRD patterns for compound C are in fair agreement with that in ICCD Card No.40–235 for CaSeO_4_∙2H_2_O. On the other hand, the XRD data for three compounds A, B, and D, that is, CaSe_2_O_7_, CaSeO_4_ and Ca_2_SeO_4_(OH)_2_ are not listed in the ICCD Cards.

**Figure 6:**
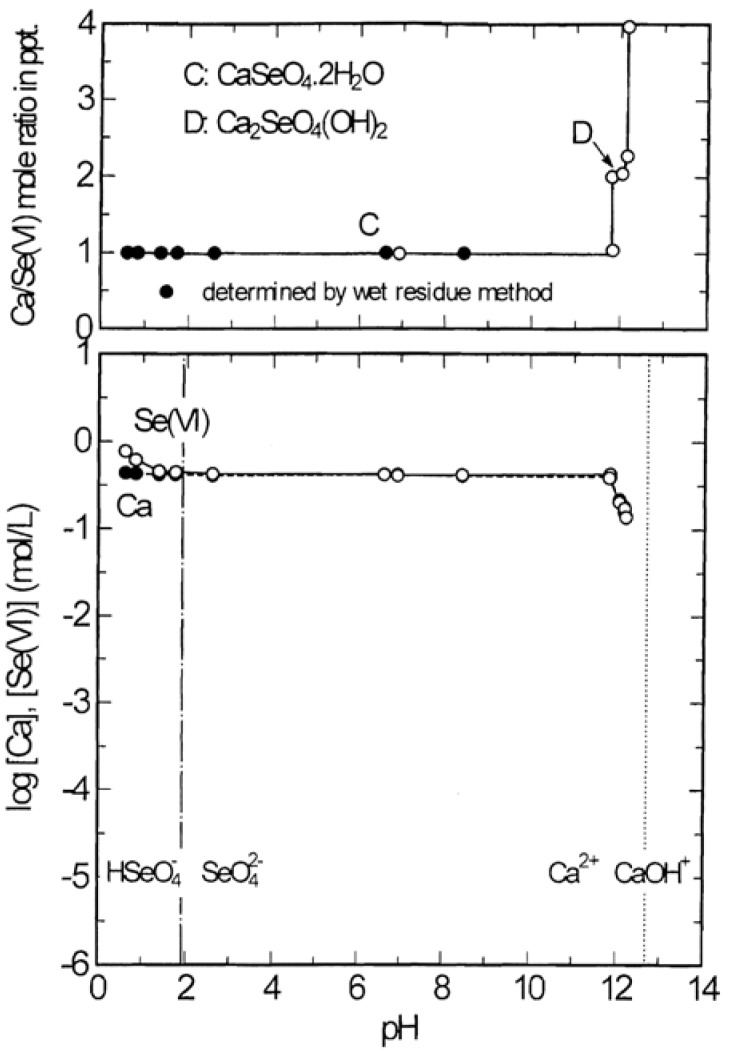
Stability and solubility regions for calcium selenates as a function of pH at 25 °C.

Figure 6 shows the stability and solubility regions for calcium selenates as function of pH at 25 °C. It is noted from Figure 6 (a) that CaSeO4∙2H2O having Ca/Se(VI) mole ratio of 1.0 is stable over the wide range of pH, while Ca_2_SeO_4_(OH) having Ca/Se(VI) mole ratio of 2.0 is stable in the very narrow range of pH 11.8 to 12.2. Also, this diagram shows two invariant points where CaSeO_4_∙2H_2_O and Ca_2_SeO_3_(OH)_2_ coexist at pH 12.0 and Ca_2_SeO_3_(OH)_2_ and Ca(OH)_2_∙H_2_O coexist at pH 12.2. As shown in Figure 6(b), the calcium concentration in solutions in equilibrium with CaSeO_4_.2H_2_O keeps constant at 0.42 mol/L in the pH range 0.6 to 12.0 and then decreases to 0.14 mol/L. The selenium(VI) concentration slightly decreases with increasing pH from 0.6 to 1.4 and then remains constant at same value of calcium concentration in the same pH range. A further increase in pH leads again to a decreased selenium(VI) concentration until it is reached to 11.1 g/L (0.14 mol/L).

### 2.4. Equilibrium in the Ba-Se(IV)-H_2_O and Ba-Se(VI)-H_2_O Systems [14]

A knowledge of the chemistry of the systems Ba-Se(IV)-H_2_O and Ba-Se(VI)-H_2_O is very important to the removal of selenium from wastewaters in an environmentally acceptable manner and sometimes selenium is recovered from process liquors by the precipitation of barium selenite and barium selenate. 

**Figure 7:**
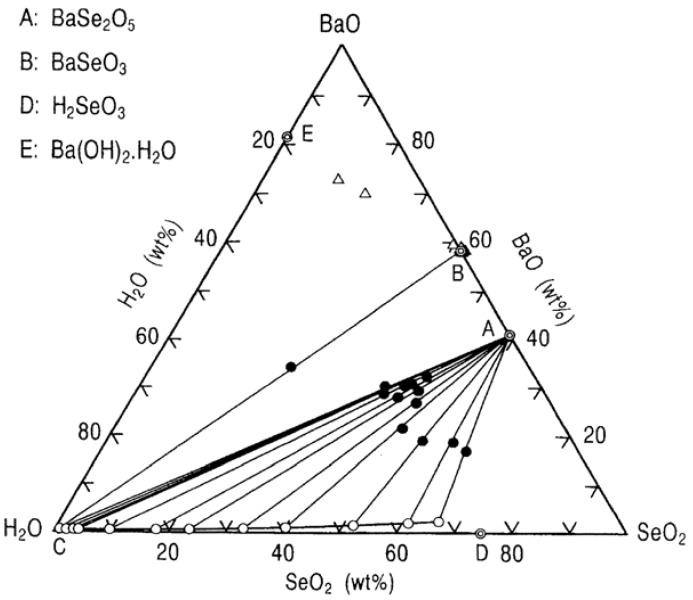
Ternary phase diagram for the BaO-SeO_2_-H_2_O system at 25 °C.

Figure 7 shows a ternary phase diagram for the BaO-SeO_3_-H_2_O system at 25 °C. In this equilibrium experiment, Ba(OH)_2_∙8H_2_O was used as the starting material. The diagram indicates the existence of two solid phases of BaSe_2_O_5_ and BaSeO_3_, given by the points A and B. At the invariant point C (BaO = 0.56 wt%, SeO_2_ = 0.83 wt%), these two solid phases are in equilibrium. Point D (SeO_2_ = 74.7 wt%) represents a saturated solution of selenious acid. 

The XRD patterns for compound A, BaSe_2_O_5_, formed in high selenious acid solutions could not be matched to any in the ICCD Cards, while the XRD patterns for compound B formed in low selenious acid solution were identical to that in ICCD Card No.8 – 268 for BaSeO_3_.

**Figure 8:**
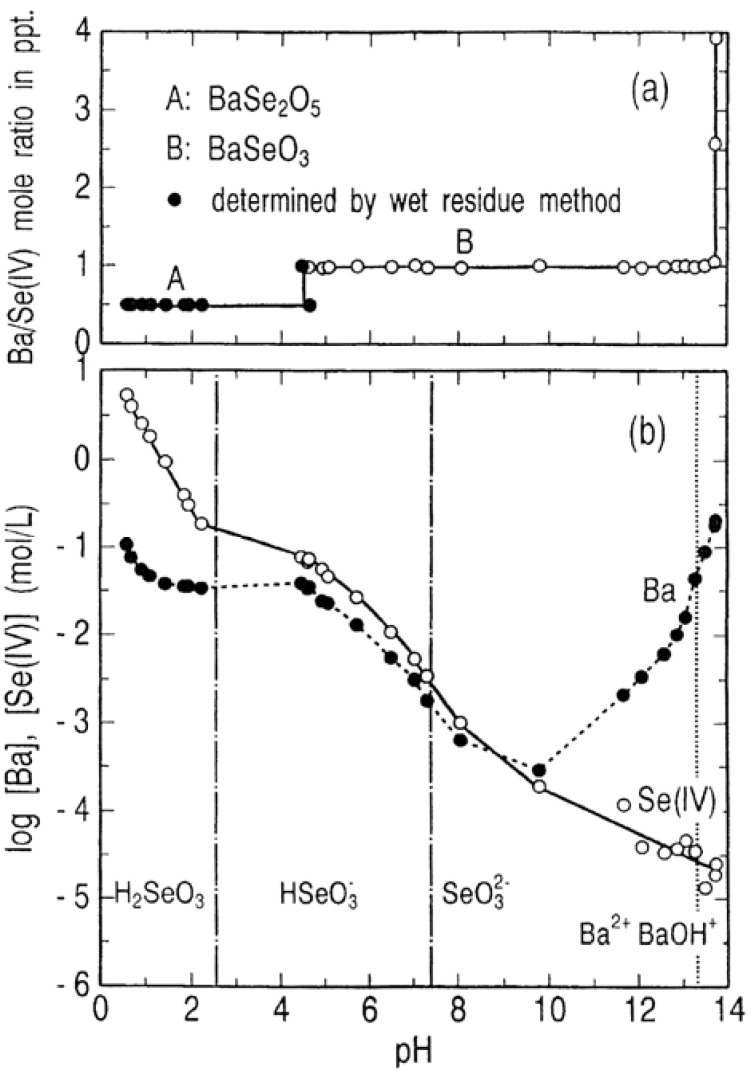
Stability and solubility regions for barium selenites as a function of pH at 25 °C.

The stability and solubility regions for barium selenites as a function of pH at 25 °C are shown in Figure 8. The plot in Figure 8(a) indicates that BaSe_2_O_5_ having Ba/Se(IV) mole ratio = 1/2 is stable at pH below 4.6 and BaSeO_3_ having Ba/Se(IV) = 1/1 in the pH range 4.6 to 13.7. Furthermore, the system Ba-Se(IV)-H_2_O has two invariant points at pH 4.6 where BaSe_2_O_5_ and BaSeO_3_ coexist and at pH 13.7 where BaSeO_3_ and Ba(OH)_2_.H_2_O coexist. Figure 8(b) shows that the barium concentration in solutions in equilibrium with the barium selenites has a minimum value of 2.9 × 10^−4^ mol/L at pH 9.8, whereas the selenium(IV) concentration has an extremely low value of 1.3 × 10^−4^ mol/L in the pH range 13.5 to 13.7 where BaSeO_3_ is in equilibrium with the solution of high barium concentration. It is noted that this lowest concentration for selenium (IV) (10 mg/L) is much higher than the limit of industrial wastewater regulation for selenium (0.1 mg/L).

**Figure 9:**
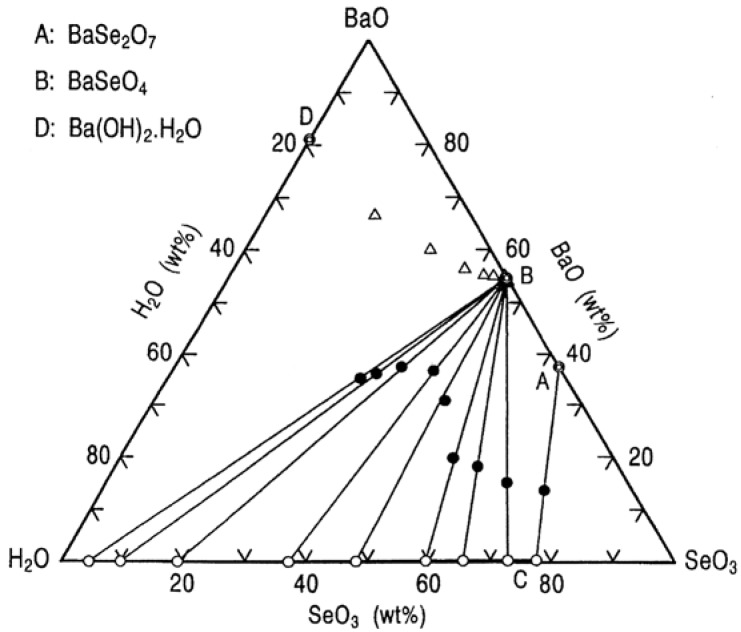
Ternary phase diagram for the BaO-SeO_3_-H_2_O system at 25 °C.

Figure 9 shows a ternary phase diagram for the system BaO-SeO_3_-H_2_O at 25 °C. It is obvious from this diagram that two compounds, indicated by A and B, can be identified. These are represented by the formulae BaSe_2_O_7_ and BaSeO_4_. At the invariant point C where the two solid phases are in equilibrium, the liquid has a low concentration of BaO between 0.40 and 0.47 wt% and a high concentration of SeO_3_ between 75.53 and 77.30 wt%. 

The XRD pattern for compound A, BaSe_2_O_7_, could not be identified in the ICCD, whereas the XRD patterns for compound B were matched with that in ICCD Card No.15–374 for BaSeO_4_.

**Figure 10:**
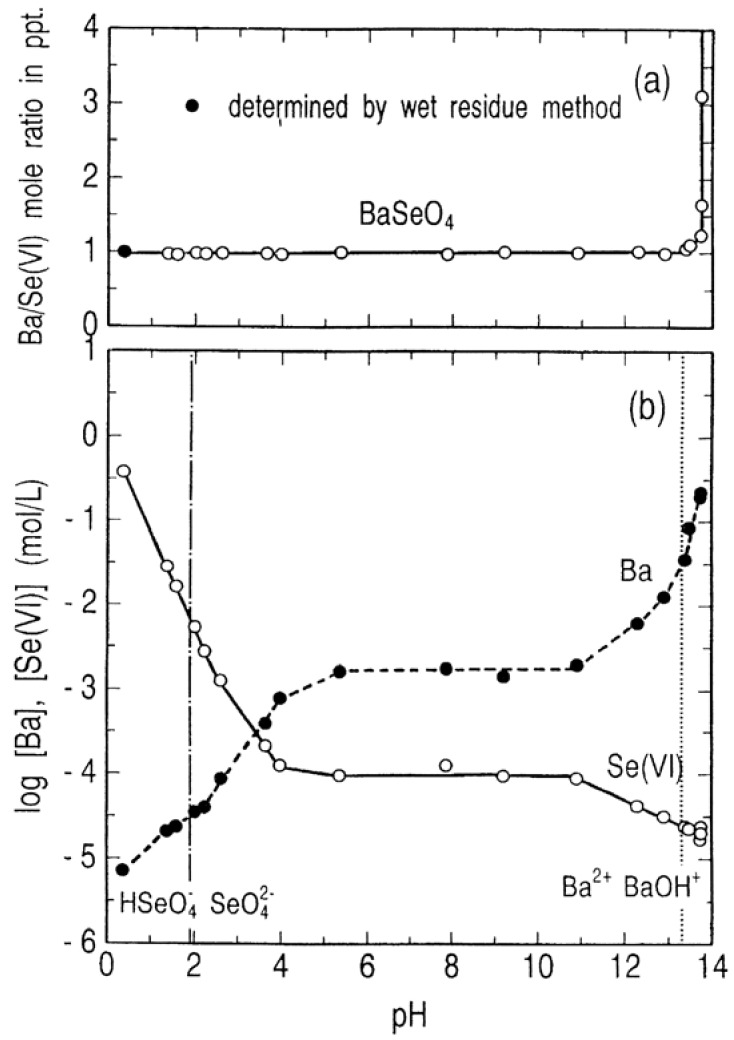
Stability and solubility regions for barium selenates as a function of pH at 25 °C.

Figure 10 shows the stability and solubility regions for barium selenates as function of pH at 25 °C. It is noted from Figure 10(a) that BaSeO_4_ having Ba/Se(VI) mole ratio = 1/1 is stable over the wide range of pH and at the invariant point of pH 13.7, BaSeO_4_ and Ba(OH)_2_∙H_2_O coexist. As shown in Figure 10(b), the barium concentration in solution in equilibrium with BaSeO_4_ increases with an increase in pH and then remains constant at 2 × 10^−3^ mol/L in the pH range 5.4 to 10.9 and increases again until the solution is saturated with Ba(OH)_2_∙H_2_O. The selenium(VI) concentration reversely decreases with an increase in pH and then remains constant at 1 × 10^−3^ mol/L in the same pH range (5.4–10.9) as well as barium. A further increase in pH leads again to a decreased selenium(VI) concentration until it is reached to about 16 mg/L. This lowest concentration of selenium (VI) is much higher than the limit of industrial wastewater regulation for selenium (0.1 mg/L).

## 3. Conditional Free Energies of Formation for Calcium Selenites and Selenates, and Barium Selenites and Selenates

Thermodynamic data are very useful for the design of the treatment of waste waters and hydrometallurgical processing. From the solubility data described above, the solubility products for calcium selenites and selenates, and barium selenites and selenates were calculated, and conditional free energies of formation were estimated from the average of solubility products obtained as follows:

Δ*fG*^φ^_298.15_ CaSeO_3_∙H_2_O = −284.2 kcal/mol (−1189.1 kJ/mol)
Δ*fG*^φ^_298.15_ Ca_2_SeO_3_(OH)_2_∙2H_2_O = −556.9 kcal/mol (−2330.1 kJ/mol).
Δ*fG*^φ^_298.15_ CaSeO_4_∙2H_2_O = −352.7 kcal/mol (−1475.6 kJ/mol)
Δ*fG*^φ^_298.15_ Ca_2_SeO_4_(OH)_2_ = −453.1 kcal/mol (−1895.9 kJ/mol)
Δ*fG*^φ^_298.15_ BaSeO_3_= −230.6 kcal/mol (−964.7 kJ/mol).
Δ*fG*^φ^_298.15_ BaSeO_4_= −249.0 kcal/mol (−1041.8 kJ/mol).

They are not standard state values because there is no allowance for Ca-Se(IV,VI) species or Ba-Se(VI,VI) complexes in the thermodynamic calculation; however, they may be available as conditional values. Here, the solubility product constant for Fe_2_(SeO_3_)_3_∙5H_2_O cannot be calculated, as thermodynamic data for the ferric selenite complex as FeHSeO_3_^2+^ proposed by Hamada [18], which is very stable in an aqueous solution containing ferric ion [15], is lacking. Also, the solubility product constant for calcium selenites and selenates, and barium selenites which exist in high acid solutions cannot be treated in the same way as the low solubility compounds. 

## 4. Removal of Selenium from Industrial Waste Water

For protection of the environment from pollution, it has been of the greatest interest to engineers treating selenium-bearing materials to remove selenium from process liquors of high selenium concentration and industrial effluents of low selenium concentration to a value lower than the limit set by industrial waste water regulations (0.1 mg/L). Many published techniques are available for the removal of selenium(IV) from waste water, but they are not very effective for selenium(VI) or costly. In this paper, the removal of selenium(IV) and selenium(VI) from aqueous solution (Se < 1,500 mg/L) containing sulfuric acid by the following three-stage process has been proposed based on the experimental results.

1. Removal of selenium(IV) as ferric selenite and sulfate ion as calcium sulfate
2. Removal of selenium(VI) as barium selenate and sulfate ion as barium sulfate
3. Removal of selenium(VI) by reduction-precipitation of selenium(VI) to elemental selenium with ferrous hydroxide or by adsorption of selenium(VI) on the polyamine-type weakly basic ion exchange resin (Eporasu K-6).

### 4.1. Removal of Selenium with Ferric Species [15]

**Figure 11:**
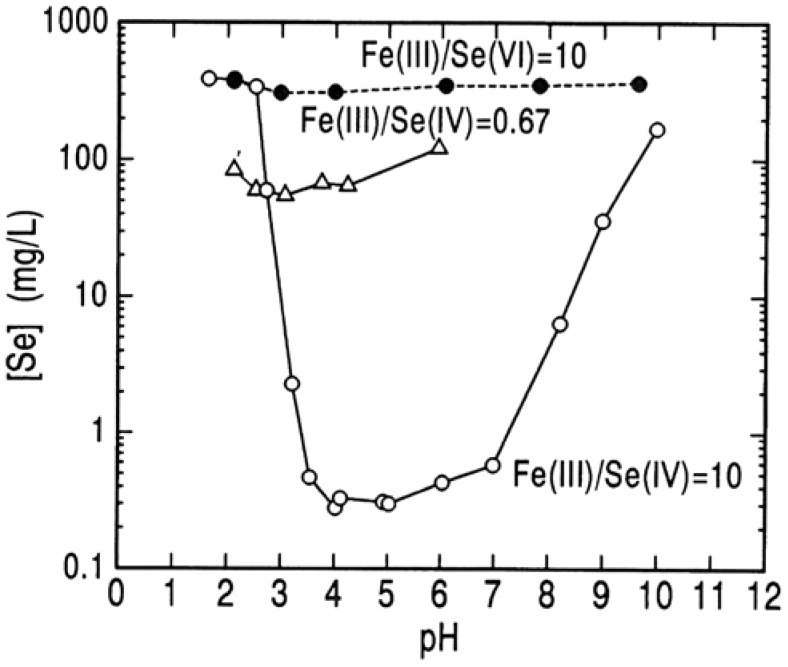
Removal of selenium(IV) and selenium(VI) by precipitation with ferric species at different pH. [Se]_o_ = 0.005 M ( = 395 mg/L); 25 °C; 2 hours.

The residual concentration of selenium(IV) in solution is shown against the solution pH in Figure 11. In this figure, the residual concentration of selenium(VI) in solution after neutralizing the Na_2_SeO_4_-Fe_2_(SO_4_)_3_ solution is also shown. At a mole ratio of Fe(III)/Se(IV) of 0.67 corresponding to the theoretical ratio for Fe_2_(SeO_3_)_3_·5H_2_O, the residual concentration of selenium(IV) in the solution has a minimum value of 55 mg/L at pH 3.1. At Fe(III)/Se(IV) mole ratio in the starting solution of 10, the concentration of selenium(IV) is reduced to a level around 0.3 mg/L in the pH range 3.5 to 7.0 by the coprecipitation with ferric hydroxide. In reverse at below pH of 2.9, the residual concentration of selenium(IV) in the solution at a mole ratio of Fe(III)/Se(IV) of 10 is higher than that at a mole ratio of 0.67. This indicates that the dissolved ferric selenite complex is very stable in an aqueous solution containing a large amount of ferric ion. On the other hand, the concentration of selenium(VI) is reduced a very little at pH range 3.0 to 4.0 even at very high mole ratio of Fe(III)/Se(VI) of 10. This means that there is not ferric selenate compound in the Fe(III)-Se(VI)-H_2_O system at moderate conditions. Hence, selenium(VI) in waste water must be removed by other techniques than the precipitation with ferric hydroxide.

### 4.2. Removal of Selenium as Barium Selenate [15]

**Figure 12:**
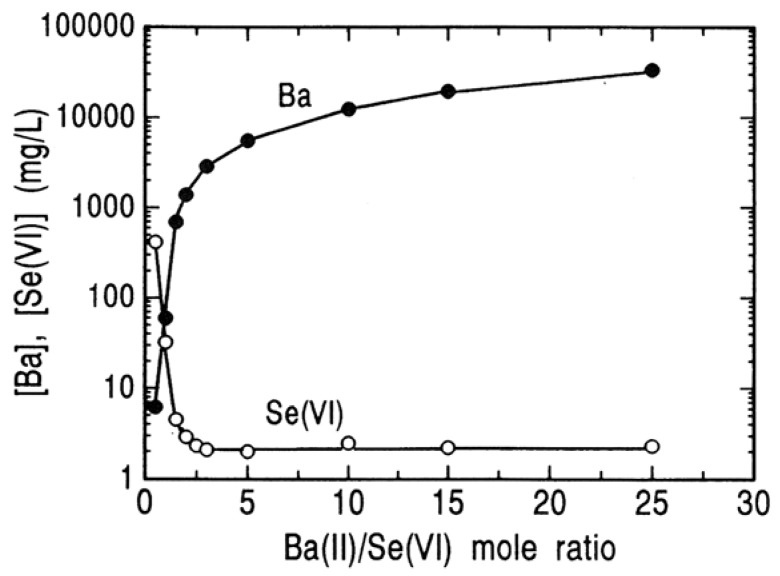
Residual concentrations of barium and selenium(VI) after precipitation of BaSO_4_ at different mole ratio of Ba(II)/Se(VI) in the starting solution. [Se(VI)]_o_ = 0.01 M; pH = 6; 25 °C; 24 hours.

In order to determine the limit of the removal of selenium(VI) by the BaSeO_4_ method, the effect of Ba/Se(VI) mole ratio in the starting solution on the removal of selenium(VI) at pH 6 was determined, and is shown in Figure 12, in which the initial concentration of selenium(VI) was 0.01 M. An increase in Ba/Se(VI) mole ratio in the starting solution leads to a drastic decrease in concentration of selenium(VI) and a terminal concentration of selenium(VI) levels of around 2 mg/L at Ba/Se(VI) mole ratio above 2.5. It is to be noted that this concentration is still above the maximum contaminant level for selenium in waste water (0.1 mg/L) and the further removal of selenium(VI) is required.

**Figure 13:**
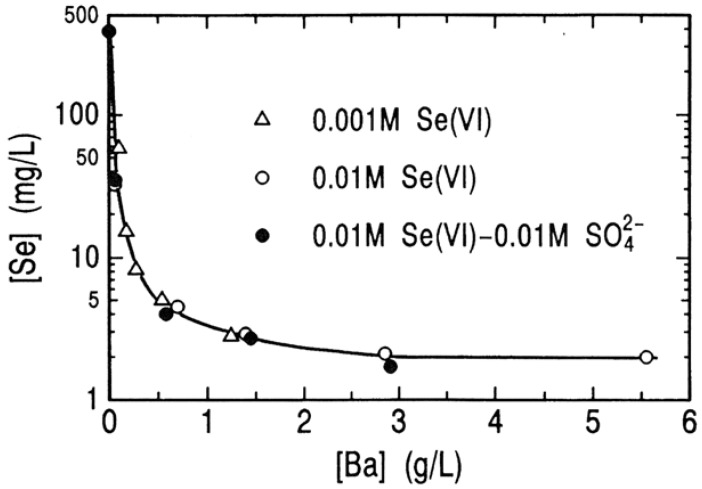
A plot of the residual selenium(VI) concentration versus the residual barium concentration after precipitation of BaSO_4_ from the solutions with and without sulfate ion. pH = 6; 25 °C; 24 hours.

The process liquors resulting from the hydrometallurgical treatments of the anode slime formed during electrolytic refining of copper usually contain large amounts of selenium and sulfuric acid. In the first stage of the process under consideration, calcium oxide is added to such solutions for adjusting pH of the solution to precipitate selenium(IV) with iron(III) species and for removing sulfate ion to a level of 0.01 M as calcium sulfate. The removal of selenium(VI) from the 0.01 M Se(VI) – 0.01 M H_2_SO_4_ solution was determined at different concentrations of residual barium, and is shown in Figure 13. In this figure, the results for the 0.001 M and 0.01 M Se(VI) solutions are also presented. It is obvious that the concentration of selenium(VI) remaining in solution is governed by the concentration of coexisting barium ion, independently of the initial concentration of selenium(VI) and the presence of sulfate ion in the starting solution.

### 4.3. Removal of Selenium(VI) by Reduction-Precipitation of Selenium(vi) to Elemental Selenium with Ferrous Hydroxide [15]

In 1988 Murphy [17] developed a new method for removing selenium(VI) from waste water based on the reduction of selenium(VI) to elemental selenium by ferrous hydroxide in alkaline solution. The stoichiometric equation in the presence of excess ferrous hydroxide is given by:

Na_2_SeO_4_ + 9Fe(OH)_2_ → Se + 3Fe_3_O_4_ + 2NaOH + 8H_2_O (1)

and magnetite is the predominant iron product. As the ratio of ferrous hydroxide to selenate decreases, maghemite is predominantly produced according to the following equation:

Na_2_SeO_4_ + 6Fe(OH)_2_ → Se + 3Fe_2_O_3_ + 2NaOH + 5H_2_O (2)

**Figure 14:**
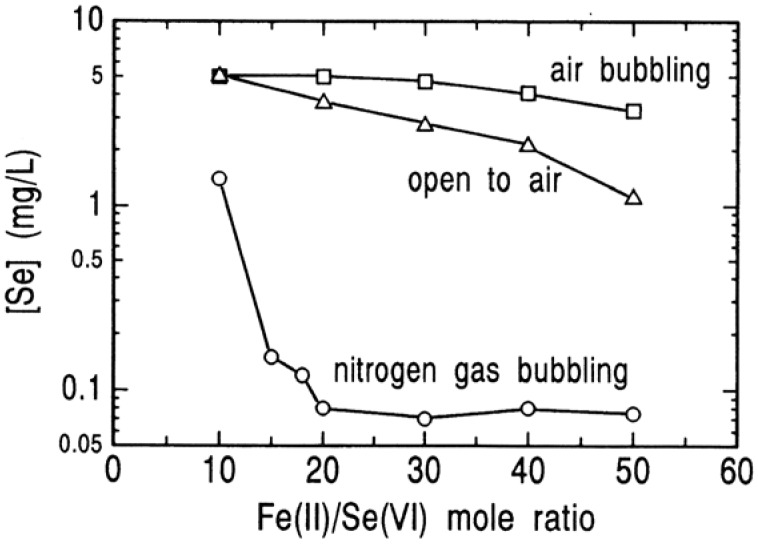
Removal of selenium(VI) by reduction with ferrous hydroxide under different atmospheres. [Se(VI)]_o_ = 5 mg/L; pH = 9.0; 70 °C; 15 min.

In 1990, the use of this method was attempted to remove selenium(VI) from precious metals tailing pond water by Lien *et al.* [19]. It was found that an addition of a large amount of ferrous sulfate corresponding to a Fe(II)/Se(VI) mole ratio of about 1,800 is required to lower the selenium(VI) concentration from 4 mg/L to below 0.1 mg/L. This large dosage of ferrous sulfate may be the result of a significant drop in the reactivity of ferrous hydroxide with dissolved oxygen from air. In our work, the reduction of selenium(VI) with ferrous hydroxide was performed in an atmosphere of nitrogen to avoid the presence of oxygen in the reaction suspension. As described above, the minimal dosage of ferrous salts holds the key of the success of the process for reduction of selenium(VI) with ferrous hydroxide. If oxygen is dissolved in the solutions, the oxidation of iron(II) would also take place in alkaline solution. In the Fe(II)/Se(VI) mole ratio range of 10 to 50, the effect of oxygen on the removal of selenium(VI) was determined at an initial selenium(VI) concentration of 5 mg/L, pH 9.0 (where selenium(VI) is reduced most favorably), 70 °C and reaction time of 15 minutes, and is shown in Figure 14. The decrease in concentration of selenium by a normal progress of the reaction of selenium(VI) with ferrous hydroxide is observed in the oxygen-free solution degassed with nitrogen, but as predicted above, the dissolved oxygen from air leads to significant suppression of removal of selenium. Specially, this effect is most remarkable when air was bubbled into the solution. This means that in the air-bubbling solution the efficient amount of ferrous hydroxide for the reduction of selenium(VI) is diminished by a rapid progress of the oxidation of iron(II) by fully supplying oxygen into the solution and consequently the reduction reaction proceeds more slowly than in the solution exposed to air.

In the removal of selenium from industrial effluents of low selenium concentration, the first and second stages in three-stage process considered in our work are not always required, if selenium(IV) contained in the effluents is effectively removed together with selenium(VI) by reduction with ferrous hydroxide. The effect of Fe(II)/Se(IV) mole ratio on the removal of selenium(IV) of an initial concentration of 50 mg/L at pH 9.0 in an atmosphere of nitrogen was determined. Here, the reaction time was 60 minutes. The reduction-precipitation of selenium(IV) to elemental selenium proceeds in the same manner at 25 °C and 70 °C, and results in a sufficiently low concentration of selenium at Fe(II)/Se(IV) mole ratio of 4. This value corresponds to the stoichiometric ratio in the reaction given by Equation (3). It should be concluded that in an atmosphere of nitrogen, selenium(IV) levels as low as 0.1 mg/L can be achieved by adding small amounts of almost stoichiometric amount of ferrous salts, independently of reduction temperature:

Na_2_SeO_3_ + 4Fe(OH)_2_ → Se + 2Fe_2_O_3_ + 2NaOH + 3H_2_O (3)

**Figure 15:**
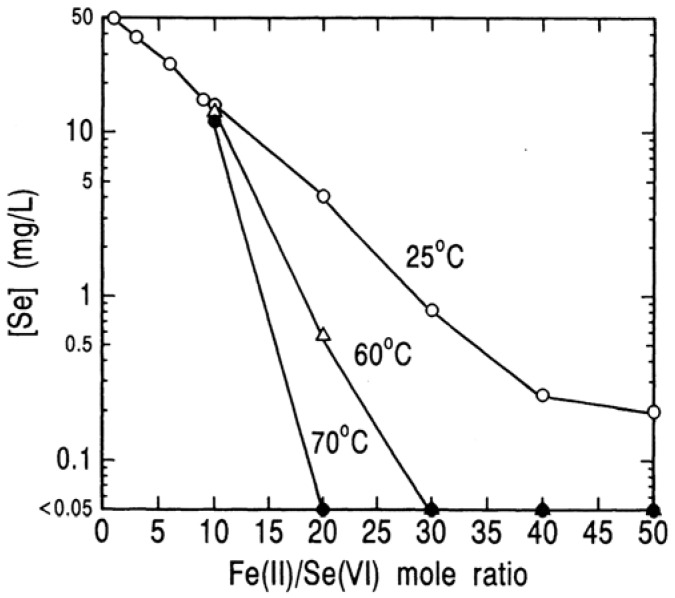
Effect of Fe(II)/Se(VI) mole ratio on removal of selenium(VI) by reduction with ferrous hydroxide in an atmosphere of nitrogen. [Se(VI)]_o_ = 50 mg/L; pH = 9.0; 70 °C; 1 hour.

In the same manner, the removal of selenium(VI) was determined, and is shown in Figure 15. Differently from selenium(IV) removal, a rise in temperature significantly accelerates the reduction of selenium(VI). At 25 °C even at a high mole ratio of Fe(II)/Se(VI) of 50 the selenium concentration of 0.1 mg/L is not attained, but at 70 °C selenium can be removed to a level lower than 0.1 mg/L at a ratio of 20. In our work, the similar trends were obtained for the removal of selenium(VI) at an initial concentration of 5 mg/L. 

### 4.4. Removal of Selenium(iv,vi) from Solution Containing Sulfuric Acid by Three-Stage Process Using by Reduction-Precipitation of Selenium(vi) to Elemental Selenium with Ferrous Hydroxide in the Third Stage [15]

The operation conditions and the main reactions for each stage in the process proposed in our work were described already. The three-stages, precipitation of ferric selenite and calcium sulfate, precipitation of barium selenate and barium sulfate and reduction-precipitation of selenium(VI) to elemental selenium with ferrous hydroxide were combined for the removal of selenium(IV,VI) from solution containing sulfuric acid. This process was modeled with the feed solution of 0.01 M ( = 790 mg/L) in Se(IV), 0.01 M ( = 790 mg/L) in Se(VI) and 10 g/L in H_2_SO_4_.

At the first stage, after 5 mL of 0.5 M Fe_2_(SO_4_)_3_ solution (corresponds to a mole ratio of Fe(III)/total Se of 0.5) was added into 500 mL of the feed solution, the solution was neutralized to pH of 3.5 with calcium hydroxide and held with stirring at 25 °C for one hour. The typical analytical data for solutions of six runs are listed in Table 1, which indicates that the majority of selenium(IV) is removed as ferric selenite and a small amount of selenium(VI) is also removed. Sulfate ion may be removed to a level of 10^−1.6^ mol/L (= 2.5 g/L), calculated from data for calcium concentration (0.5 g/L) and solubility product of 10^−4.4^ for calcium sulfate.

At the second stage, 1.0 M BaCl_2_ solution of various volumes was added into the filtrates containing selenium(IV) of 10 mg/L and selenium(VI) of 740 mg/L from the first stage and the pH of solutions was adjusted to 6 and held with stirring at 25 °C for two hours. As shown in Table 2, selenium(IV) remaining in the solution from the first stage solution can be removed to a very low level as barium selenite (BaSeO_3_) by addition of a large excess of BaCl_2_. Selenium(VI) can be removed to a level of 100 mg/L when the barium concentration in solution was kept at about 50 mg/L. Furthermore, for removing selenium(VI) to a level of 10 mg/L the residual concentration of barium is required to be about 500 mg/L. 

At the third stage, the two filtrates containing 4.7 mg/L and 51 mg/L selenium from the second stage were treated by dissolving ferrous sulfate of weights corresponding to mole ratios of 50 at 25 °C and 20 at 70 °C in an atmosphere of nitrogen and then by adjusting pH of solution to 9.0. Table 3 shows the analytical data for solutions after the reduction-precipitation with ferrous hydroxide for one hour. In both cases, the reduction of selenium(VI) as well as selenium(IV) with ferrous hydroxide is possible to remove selenium to below 0.1 g/L from the liquor from the second stage except the case which the filtrate containing 51 mg/L selenium(VI) was treated at Fe(II)/Se(VI) mole ratio of 50 and at 25 °C. Also, barium is precipitated with sulfate ion from ferrous sulfate added. 

## 5. Adsorption of Selenium(VI) on Polyamine – Type Weakly Basic Ion Exchange Resin [16]

### 5.1. Adsorption of Selenium(VI) in Batch Experiments 

**Figure 16:**
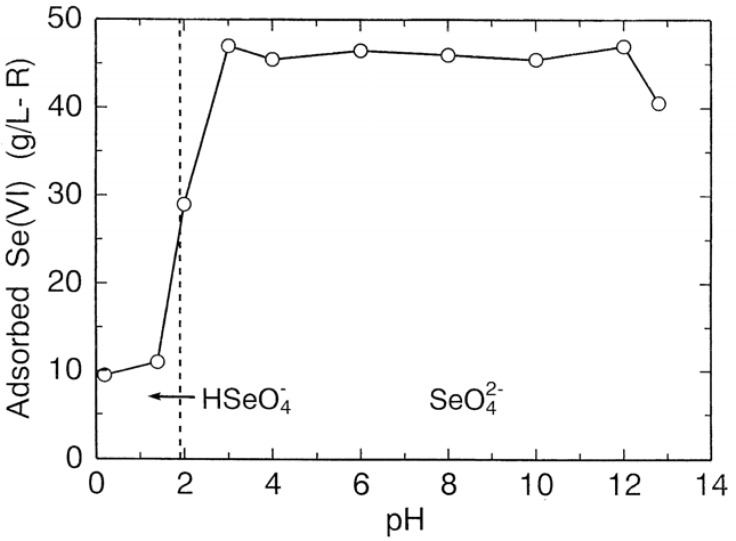
Effect of pH on adsorption of selenium(VI) on resin.

The adsorption capacity of weakly basic ion exchange resin is generally affected by hydroxyl ion in solution. The adsorption capacity of the resin (Eporasu K-6) was determined as a function of pH for selenium(VI) by batch experiments, and is shown in Figure 16. Here, selenium(VI) concentration in the starting solution was 60 mg/L. The amount of selenium(VI) adsorbed by the resin jumps up as soon as a stable species in solution changes from HSeO_4_^−^ to SeO_4_^2−^ by a rise in pH and has a high value of 46 g/L-R over a wide pH range of 3 to 12. At pH 13 a decrease in adsorption is observed. This may be attributed to the depression of ionization of the functional group of the resin due to a large amount of hydroxyl ion in solution. From these results, it is noted that selenium(VI) can be effectively absorbed on the resin over a wide pH range than selenium(IV). 

**Figure 17:**
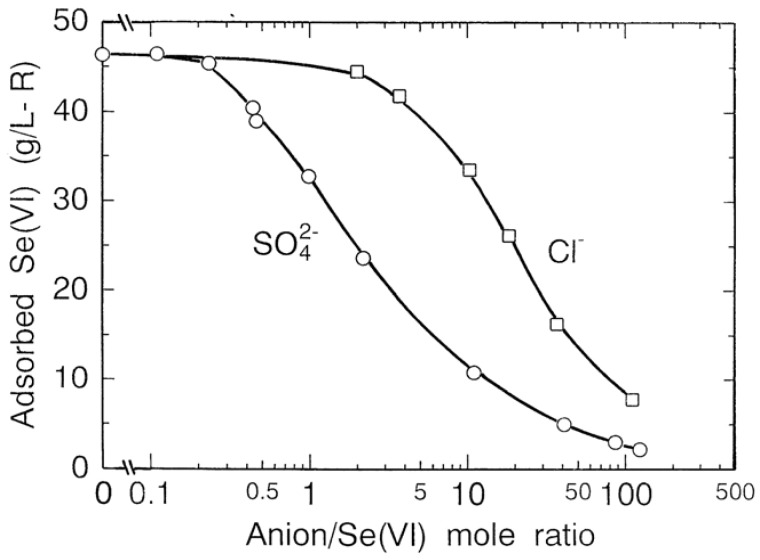
Effect of coexisting anion on adsorption of selenium(VI) on resin.

Waste waters often contain significant quantities of various anions such as SO_4_^2−^and Cl^−^. Specially, since SO_4_^2−^ has chemical similarity to SeO_4_^2−^, a competitive adsorption reaction may take place between SeO_4_^2−^ and SO_4_^2−^. The influence of SO_4_^2−^and Cl^−^ on the adsorption of selenium(VI) on the resin was determined by adding various amounts of Na_2_SO_4_ or NaCl to the selenium(VI) solution of 60 mg/L at pH 6.0, and is shown in Figure 17. In the coexistence of SO_4_^2−^, the amount of selenium(VI) adsorbed on the resin starts to decrease from SO_4_^2−^/SeO_4_^2−^ mole ratio of 0.3 and shows a decrease of 50 percent at mole ratio of 2. In the coexistence of Cl^−^, the amount of selenium(VI) adsorbed starts to decrease at Cl^−^/SeO_4_^2−^ mole ratio of 3 and shows a decrease of 50 percent at mole ratio of 20. These indicate that the adsorption of the resin is in order of SeO_4_^2−^ > SO_4_^2−^ ≥ Cl^−^, but SO_4_^2−^ in waste water must be removed prior to use of the resin for removing SeO_4_^2−^ because of competitive adsorption.

### 5.2. Adsorption of Se(VI) in Column Experiments

**Figure 18:**
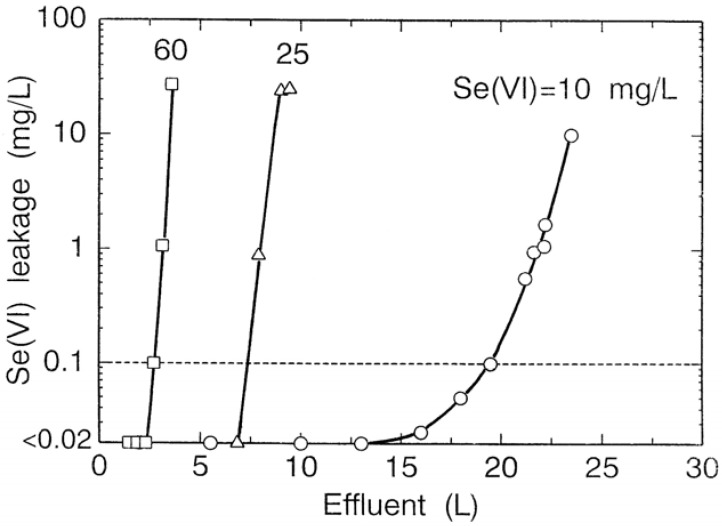
Breakthrough curves of selenium(VI) at different concentration of selenium(VI). pH = 6.0; bed volume of resin = 5.0 mL; SV = 10.

Selenium(VI) concentrations of 10, 25 and 60 mg/L in the feed solution (pH = 6.0) were passed through the resin bed (7.6 mmφ × 110 mm) packed in the column at space velocities of 10. As shown in Figure 18, after selenium(VI) starts to be detected in the effluent, the selenium(VI) concentration in effluent leaked out curves gently up with an increase in effluent volume at selenium(VI) concentration of 10 mg /L in the feed solution, while jumps sharply up at both 25 and 60 mg/L. The amount of selenium(VI) adsorbed on the resin calculated from a permissible effluent volume for disposal is 65.1 g/L-R for selenium(VI) concentration of 10 mg /L in the feed solution, 62.5 g/L-R for 25 mg/L and 54.4 g/L-R for 60 mg/L, which indicates that it is not advisable to use the resin for removing a high level of selenium(VI).

In order to find a suitable eluent of selenium(VI) absorbed, batch experiments were carried out for hydrochloric acid and sodium hydroxide solutions of various concentrations. Here, the resin loaded by selenium(VI) at 46 g/L-R was contacted with the eluents for three hours. As shown in Figure 18, in hydrochloric acid solution the amount of selenium(IV) adsorbed by the resin decreases sharply to 6g/L-R with a slight increase in acidity and then still continues to decrease marginally as the acidity increases. In sodium hydroxide solution, the amount of selenium(IV) adsorbed decreases gradually with an increase in basicity and then stays at around 13 g/L-R over NaOH concentration range above 1.0 mol/L. These suggests that hydrochloric acid solution will acts more effectively as an eluent of selenium(VI) from the resin than sodium hydroxide solution. 

The cycles of adsorption and elution of selenium(VI) were repeated three times. A decrease in loading capacity caused by recycling the resin was not observed and selenium(VI) was eluted from the resin with relatively small volumes of 1 M HCl (22 L/L-R), which indicate that the resin can be used repeatedly for the removal of selenium(VI) from waste water.

### 5.3. Removal of Selenium(IV,VI) from Solution Containing Sulfuric Acid by Three-Stage Process Using by Adsorption of Selenium(VI) on the Polyamine-Type Weakly Basic Ion Exchange Resin (Eporasu K-6) in the Third Stage 

**Figure 19:**
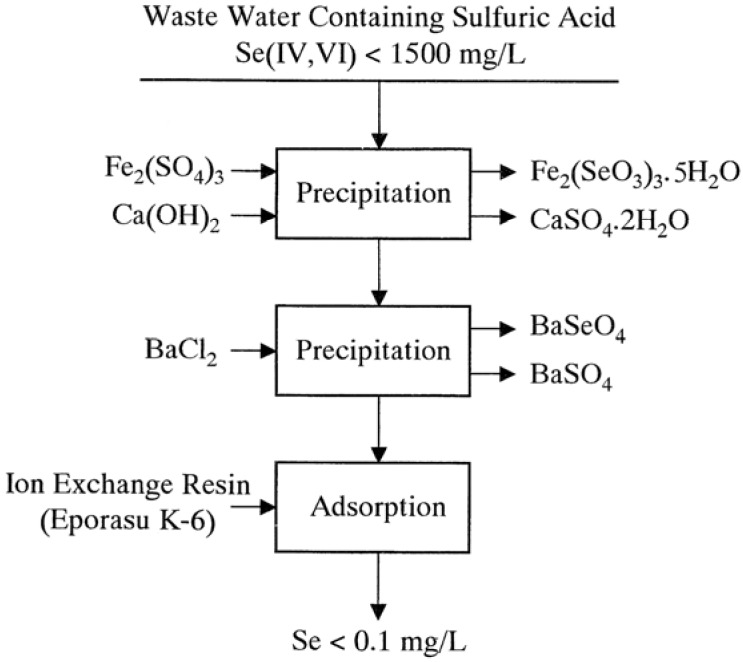
Flowsheet of process for removal of selenium from industrial waste water.

As noted previously, in the third stage, the remaining selenium(VI) is removed to a lower concentration than 0.1 mg/L by reduction-precipitation of selenium(VI) to elemental selenium with ferrous hydroxide at pH 9 at 70 °C in an atmosphere of nitrogen. However, this process requires very strict conditions to effectively remove selenium(VI). Replacement of the ferrous hydroxide reduction process with the adsorption process using the polyamine-type weakly basic ion exchange resin (Eporasu K-6) yields a simplified selenium removal process which is shown in Figure 19. This process seems to be promising to remove selenium from high concentration levels of selenium(IV) and selenium(VI) in acidic sulfate waste water to below 0.1 mg/L.

## 6. Conclusions

In this paper, the chemistry of the M (M = Fe, Ca, Ba)-Se-H_2_O systems at 25 °C was described and the process for the removal of selenium from the industrial waste water was proposed. In order to develop a simpler and easier process, the compounds of lower solubility or the other reactions should be determined by further investigations.

## Figures and Tables

**Table 1:** Typical analysis of filtrate from the first stage (mg/L).

Total Se	Se(IV)	Se(VI)	Fe(III)	Ca
754	11	743	1.4	595

**Table 2:** Analysis of filtrates from the second stage.

BaCl_2_ added(mol/L)	Total Se(mg/L)	Se(IV)(mg/L)	Se(VI)(mg/L)	Ba(mg/L)
0.03	414	4.3	410	< 0.02
0.035	102	0.35	102	43
0.035	51	0.27	51	106
0.04	8.9	0.18	8.7	608
0.045	4.7	0.05	4.7	1150
0.05	3.4	< 0.05	3.4	1860
0.075	1.7	< 0.05	1.7	5180

**Table 3:** Analysis of filtrates from the third stage (mg/L).

Condition	Total Se	Se(IV)	Se(VI)	Ba	Fe
Feed	4.7	0.05	4.7	1150	< 0.02
Fe(II)/Se mole ratio = 50 & T = 25 °C	0.05	< 0.05	0.05	657	< 0.02
Fe(II)/Se mole ratio = 20 & T = 70 °C	0.06	< 0.05	0.06	631	< 0.02
Feed	51	0.27	51	106	< 0.02
Fe(II)/Se mole ratio = 50 & T = 25 °C	0.1	< 0.05	0.1	< 0.02	3.8
Fe(II)/Se mole ratio = 20 & T = 70 °C	0.05	< 0.05	0.05	< 0.02	< 0.02
